# Bronquiectasias no relacionadas con fibrosis quística en pediatría: perfil de una cohorte de pacientes con errores innatos de la inmunidad en un centro de referencia de Cali, Colombia

**DOI:** 10.7705/biomedica.7558

**Published:** 2024-12-23

**Authors:** Andrea Murillo, Darly Marín, Jacobo Triviño, Oriana Arias, Diana Duarte, Paola Pérez, Jaime Patiño, Harry Pachajoa, Diego Medina, Alexis Franco, Manuela Olaya-Hernández

**Affiliations:** 1 Facultad de Ciencias de la Salud, Departamento de Medicina, Universidad ICESI, Cali, Colombia Universidad ICESI Facultad de Ciencias de la Salud Departamento de Medicina Universidad ICESI Cali Colombia; 2 Centro de Investigaciones Clínicas, Fundación Valle del Lili, Cali, Colombia Fundación Valle del Lili Centro de Investigaciones Clínicas Fundación Valle del Lili Cali Colombia; 3 Servicio de Alergología e Inmunología Pediátrica, Departamento de Pediatría, Fundación Valle del Lili, Cali, Colombia Fundación Valle del Lili Servicio de Alergología e Inmunología Pediátrica Departamento de Pediatría Fundación Valle del Lili Cali Colombia; 4 Servicio de Neumología Pediátrica, Departamento de Pediatría, Fundación Valle del Lili, Cali, Colombia Fundación Valle del Lili Servicio de Neumología Pediátrica Departamento de Pediatría Fundación Valle del Lili Cali Colombia; 5 Servicio de Infectología Pediátrica, Departamento de Pediatría, Fundación Valle del Lili, Cali Colombia Fundación Valle del Lili Servicio de Infectología Pediátrica Departamento de Pediatría Fundación Valle del Lili Cali Colombia; 6 Servicio de Hemato-oncología Pediátrica, Departamento de Pediatría, Fundación Valle del Lili, Cali Colombia Fundación Valle del Lili Servicio de Hemato-oncología Pediátrica Departamento de Pediatría Fundación Valle del Lili Cali Colombia

**Keywords:** bronquiectasia, pediatría., Bronchiectasis, pediatrics.

## Abstract

**Introducción.:**

Los errores innatos de la inmunidad se asocian frecuentemente con bronquiectasias. Actualmente, el diagnóstico de los errores innatos de la inmunidad ha mejorado porque se conoce con certeza la asociación de estas entidades con el daño progresivo de las vías respiratorias. Esto ha permitido el reconocimiento y la intervención adecuada, lo cual reduce el deterioro de la función pulmonar y mejora la calidad de vida.

**Objetivo.:**

Describir un grupo de pacientes con bronquiectasias no relacionadas con la fibrosis quística y con diagnóstico de errores innatos de la inmunidad, estudiados en un centro de referencia de inmunología en Cali, Colombia.

**Materiales y métodos.:**

Se desarrolló un estudio observacional, descriptivo y retrospectivo de pacientes menores de 18 años con diagnóstico de errores innatos de la inmunidad y bronquiectasias no relacionadas con fibrosis quística, entre diciembre de 2013 y diciembre de 2023 en la Fundación Valle del Lili, en Cali (Colombia).

**Resultados.:**

Se incluyeron 17 pacientes con diagnóstico de bronquiectasias no relacionadas con fibrosis quística y errores innatos de la inmunidad, cuya edad media fue de nueve años. El inferior fue el lóbulo pulmonar más frecuentemente afectado y su compromiso fue unilateral en la mayoría de los casos. La inmunodeficiencia con predominio de defectos de los anticuerpos fue la más común, seguida de las inmunodeficiencias combinadas asociadas con síndromes. Trece pacientes presentaron compromiso de la inmunidad humoral y 4 pacientes, alteraciones en la inmunidad humoral y celular. En 12 pacientes se identificaron modificaciones genéticas relacionadas con su fenotipo. Trece pacientes recibieron suplemento de inmunoglobulina intravenosa y 3 fallecieron.

**Conclusión.:**

La inmunodeficiencia con predominio de defectos de los anticuerpos, seguida de las inmunodeficiencias combinadas asociadas con características sindrómicas, fueron los errores innatos de la inmunidad que con mayor frecuencia se acompañaron de bronquiectasias no relacionadas con la fibrosis quística.

## Abstract (en)

**Introduction.:**

Inborn errors of immunity are frequently associated with bronchiectasis. The diagnostic performance of these inborn errors has improved because the association of some of these entities with progressive airway damage is better known. This knowledge has allowed recognition and appropriate intervention reducing deterioration of the pulmonary function and improving quality of life.

**Objective.:**

To describe a group of patients with bronchiectasis not related to cystic fibrosis who were diagnosed with inborn errors of immunity and have been studied in an immunology reference center in Colombia.

**Materials and methods.:**

We conducted an observational, descriptive, and retrospective study with participating patients under 18 years, diagnosed with inborn errors of immunity and non-cystic fibrosis bronchiectasis, between December 2013 and December 2023 at the Fundación Valle del Lili in Cali, Colombia.

**Results.:**

Seventeen patients were diagnosed with non-cystic fibrosis bronchiectasis and inborn errors of immunity. Their mean age was nine years. The lower pulmonary lobe was the most frequently affected segment, and in most cases, unilaterally. The most prevalent alteration was predominantly antibody inmunodeficiency, followed by combined immunodeficiencies associated with syndromes. Thirteen patients had humoral immunity compromise, while 4 exhibited humoral and cellular immunity alterations. Additionally, 12 patients presented genetic mutations related to their phenotype. Thirteen patients, underwent supplementation with intravenous immunoglobulin, and 3 died.

**Conclusion.:**

The inborn errors of immunity most frequently associated with non-cystic fibrosis bronchiectasis, were predominantly antibody deficiency and combined immunodeficiencies with syndromic features.

Las bronquiectasias son dilataciones permanentes de la vía aérea, que se manifiestan con tos crónica, infecciones respiratorias pulmonares recurrentes, exacerbaciones, deterioro de la función pulmonar y un impacto negativo de la calidad de vida ^1^^,^^2^. Los errores innatos de la inmunidad están asociados con el desarrollo del 12 al 34 % de las bronquiectasias no relacionadas con la fibrosis quística ^1^^,^^3^.

Los errores innatos de la inmunidad son un grupo heterogéneo de enfermedades raras atribuidas a más de 400 errores genéticos que afectan diferentes componentes del sistema inmunológico innato y adaptativo, y que resultan en una mayor susceptibilidad a infecciones, autoinmunidad, atopia, desregulación inmunológica y neoplasias malignas. De acuerdo con la clasificación del 2022 de la *International Union of Immunological Societies* (IUIS) ^4^, se describen 10 grupos con base en el componente del sistema inmunológico afectado.

Los errores innatos de la inmunidad relacionados con bronquiectasias pueden constituir un reto diagnóstico por su heterogeneidad, presentación clínica y edad de aparición. Para llegar a un diagnóstico definitivo, generalmente se requiere un enfoque multidisciplinario y estudios genéticos ^1^. Lo anterior puede contribuir a retrasar el tratamiento específico, lo que afecta negativamente la función pulmonar, debido al progreso de las bronquiectasias, deteriorándose la calidad de vida del paciente y, en muchos casos, empeorando el pronóstico de la enfermedad de base ^1^^,^^5^.

Los errores innatos de la inmunidad que cursan con deficiencias de anticuerpos han sido los más asociados con bronquiectasias ^1^^,^^6^. Estos pacientes presentan desregulación inmunológica y limitaciones para controlar infecciones sino-pulmonares, y son susceptibles a infecciones por gérmenes encapsulados, como *Streptococcus pneumoniae* y *Haemophilus influenzae*, sucesos que podrían explicar la gran frecuencia de desarrollo de bronquiectasias ^1^^,^^6^^,^^7^. Además, el espectro clínico de los errores innatos de la inmunidad que cursan con deficiencias de anticuerpos es amplio; comprende desde compromisos leves, como la deficiencia selectiva de IgA y de subclases de IgG (IgG1, IgG2, IgG3, IgG4) con niveles normales de IgG totales, y la deficiencia de anticuerpos específicos con niveles normales de IgG, IgAe IgM, hasta alteraciones graves, como la agammaglobulinemia ligada al cromosoma X y la inmunodeficiencia combinada grave ^6^^,^^8^^-^^10^.

El error innato de la inmunidad más relacionado y estudiado en pacientes con bronquiectasias es la inmunodeficiencia común variable, dado que es la inmunodeficiencia humoral grave más frecuente ^6^^,^^8^. Esta cursa con disminución o ausencia de IgG, IgA o IgM; producción ausente o disminuida de anticuerpos específicos contra diferentes antígenos, y desregulación inmunológica. Sus manifestaciones clínicas incluyen: infecciones recurrentes, enfermedades autoinmunitarias, alergias respiratorias, neoplasias linfoides e inflamación granulomatosa ^8^^,^^10^. Sin embargo, cualquier error innato de la inmunidad puede cursar con bronquiectasias, como la inmunodeficiencia combinada grave, las inmunodeficiencias combinadas con características sindrómicas, o las alteraciones en la regulación inmunológica ^8^. La ganancia de función del STAT3, el síndrome de hiper-IgE (por pérdida de función de STAT3, DOCK8 o deficiencia de TYK2), el aumento de función de la fosfatidilinositol-3-cinasa, la haploinsuficiencia de CTLA-4, la deficiencia de LRBAy la enfermedad granulomatosa crónica, entre otras, también se han relacionado con bronquiectasias ^1^^,^^5^^,^^8^^,^^11^.

La tomografía de alta resolución del tórax (TACAR) es el estudio de referencia para el diagnóstico de bronquiectasias, pues permite una mejor visualización de la dilatación bronquial en el tejido pulmonar distal, evalúa el aumento de la relación arteria-bronquio ^2^^,^^8^, e identifica el engrasamiento de la pared bronquial y la falta de disminución del calibre de los bronquios desde el centro hacia la periferia. También, permite observar las estructuras bronquiales en la periferia pulmonar y la hipoperfusión en mosaico ^12^^,^^13^.

Según algunos estudios, en los pacientes con errores innatos de la inmunidad, las bronquiectasias afectan frecuentemente múltiples lóbulos pulmonares ^1^^,^^14^^,^^15^. También se ha descrito que aquellas localizadas en los lóbulos inferiores se relacionan con inmunodeficiencia común variable ^6^; sin embargo, esto no es exclusivo de esta entidad clínica, pues las bronquiectasias pueden presentarse en otras enfermedades diferentes a los errores de la inmunidad, como la discinesia ciliar primaria y la aspiración crónica ^1^^,^^12^^,^^14^.

En Colombia, se desconoce la incidencia de bronquiectasias en pacientes pediátricos con errores innatos de la inmunidad. En un reporte de Bogotá, se describió un 10 % de bronquiectasias en una población con errores innatos de la inmunidad y manifestaciones pulmonares, sin caracterización inmunológica ^16^.

El objetivo del presente estudio fue describir un grupo de pacientes pediátricos con bronquiectasias no relacionadas con fibrosis quística y con diagnóstico de errores innatos de la inmunidad, estudiados en un centro de referencia de inmunología en el suroccidente de Colombia.

## Materiales y métodos

Se trata de un estudio observacional, descriptivo y retrospectivo, que incluyó pacientes menores de 18 años con diagnóstico de errores innatos de la inmunidad y presencia de bronquiectasias no relacionadas con fibrosis quística, entre diciembre de 2013 y diciembre de 2023 en la Fundación Valle del Lili. Se excluyeron aquellos pacientes con diagnóstico de inmunodeficiencia secundaria o adquirida.

El diagnóstico de errores innatos de la inmunidad se basó en la clasificación de la *International Union of Immunological Societies* (IUIS) de 2022. Se hizo el diagnóstico según los criterios establecidos para cada enfermedad por el registro de la *European Society for Immunodeficiencies* (ESID), consignados en el documento “Working definitions for clinical diagnosis of PID”, publicado en el 2019 ^17^.

El equipo investigador revisó los registros clínicos institucionales de cada paciente para, posteriormente, recopilar las variables seleccionadas en la base de datos, creada en la plataforma E-REDCap. Entre las variables se encontraban datos demográficos, características clínicas, paraclínicas, inmunológicas-subpoblaciones de células T (CD3, CD4, CD8), B, NK-e inmunoglobulinas séricas-IgG, IgM, IgA, IgE-, reacción vacunal proteica y polisacárida a 23 serotipos de neumococo, linfoproliferación ante mitógenos (fitohemaglutinina, PHA) y resultado del estado vital de los pacientes.

### Análisis estadístico

Se hizo un análisis descriptivo de las variables. Aquellas de naturaleza categórica se resumieron como frecuencias absolutas y proporciones; las cuantitativas se expresaron según la evaluación de su distribución (paramétrica o no), calculada mediante la prueba de Shapiro-Wilk, con su respectiva medida de tendencia central y de dispersión. Los análisis se hicieron con el *software* R Studio, versión 4.3.3.

### Consideraciones éticas

Este estudio fue evaluado y aprobado por el Comité de Ética e Investigación Biomédica de la Fundación Valle del Lili, bajo el número de aprobación 2023.217. Asimismo, los principios éticos de la Declaración de Helsinki se respetaron en todas las etapas del estudio. El comité de ética institucional consideró correcto omitir la exigencia individual del consentimiento informado.

## Resultados

De los registros clínicos de la Fundación Valle del Lili, se extrajo la información de los pacientes con diagnóstico de bronquiectasias y errores innatos de la inmunidad. Se hizo el cruce de variables para identificar a aquellos que padecieron bronquiectasias no relacionadas con fibrosis quística. De 366 pacientes con errores innatos de la inmunidad, 17 tuvieron diagnóstico de bronquiectasias no relacionadas con fibrosis quística, de los cuales 9 eran hombres. La edad media fue de nueve años, con un rango intercuartílico (RIC) de 5 a 12 (cuadro 1).

**Cuadro 1.** Características de la población (N = 17)

Diagnóstico		
	Agammaglobulinemia de Bruton	1
	Inmunodeficiencia común variable	3
	Deficiencia selectiva de IgM	1
	Deficiencia específica de anticuerpos y alteración neurológica sindromática	1
	Hipogammaglobulinemia inespecífica	1
	Deficiencia de anticuerpos específicos y esclerosis tuberosa	1
	Inmunodeficiencias combinadas no graves asociadas a síndrome de Down	2
	Ataxia telangiectasia	2
	Síndrome de hiper-IgE	2
	Síndrome de hiper-IgM	1
	Disqueratosis congénita	1
	Neumonitis intersticial linfoidea	1
Estudio genético relacionado con el fenotipo		
	No se realizó estudio	4
	Relacionado	12
	No relacionado con el fenotipo	1
Inmunoglobulina humana inespecífica intravenosa		13
Fallecidos		3

Al momento del diagnóstico, se evaluó la inmunidad humoral en todos los pacientes y, la inmunidad celular, en 15 de ellos. En 13 pacientes se documentó compromiso de la inmunidad humoral, seis pacientes mostraron compromiso de inmunidad celular y cuatro presentaron alteraciones tanto de la inmunidad humoral como de la celular, tal como se muestra en el cuadro 2.

**Cuadro 2.** Valores de los exámenes inmunológicos

Paciente	Edad (años)	Sexo	Compromiso de inmunidad humoral	IgG (mg/ dl)	IgG (mg/ dl)	IgG (mg/ dl)	IgE (UI/L)	Compromiso de inmunidad celular	CD3 (cél/ml)	CD4 (cél/ml)	CD8 (cél/ml)	CD19 (cel/ml)	IgG contra rubéola	IgG contra hepatitis B	IgG contra neumococo (23 serotipos)	Linfoproliferación con mitogénos	Subpoblaciones de linfocitos B
1	13	M	Sí	200	<40	<25	1	No	1.289	805	425	0	+	+	-	Normal	Alterada
2	15	F	Sí	270	4	833	25	Sí	888	365	478	48	-	-	-	Normal	Alterada
3	16	M	Sí	1.076	0	546	1,2	Sí	993	317	625	45	-	-	-	-	-
4	10	F	No	1.621	416	211	941	No	1.174	571	458	341	-	-	-	-	-
5	14	F	Sí	800	172	471	11,8	No	1.300	642	607	3,7	+	+	Alterado	-	Alterada
6	3	M	Sí	660	91	53	6,69	No	2.450	1.463	791	1.018	+	-	Alterado	-	Alterada
7	16	M	Sí	270	40	31	1	-	-	-	-	-	-	-	-	-	-
8	6	M	No	1.028	218	76	5,6	Sí	309	156	127	135	+	+	-	Normal	Normal
9	3	F	Sí	1.161	90	142	12.098	No	2.866	1.284	1.806	484	+	+	-	Normal	Alterada
10	16	M	Sí	1.944	344	100	17,3	Sí	978	454	458	52	+	-	Alterado	-	-
11	8	M	Sí	580	0	107	0,4	No	3.057	2.063	689	777	-	-	Alterado	-	-
12	3	F	Sí	140	129	475	2	Sí	1.148	507	598	-	-	-	-	-	-
13	10	M	No	744	99	68	17,5	Sí	788	427	341	66	-	-	-	-	-
14	3	M	Sí	607	70	108	17	No	1.927	937	853	436	+	-	Alterado	-	-
15	8	F	No	1.190	468	77	21,9	No	3.840	1.696	2.068	1.664	+	+	-	-	-
16	2	F	Sí	1.167	236	40	2,83	-	-	-	-	-	+	-	Normal	-	Normal
17	11	F	Sí	540	193	142	669	No	1.303	629	533	277	-	-	-	-	Alterada

**Figura 1.**
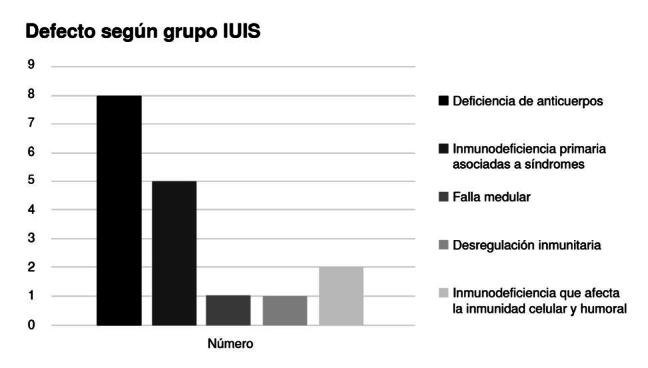
Defectos por grupo según la clasificación de la International Union of Immunological Societies (IUIS)

Según la clasificación actualizada (2022) de la IUIS (figura 1), se encontró que la inmunodeficiencia con predominio de defectos de los anticuerpos fue el error de la inmunidad más frecuente (n = 8), seguida de las inmunodeficiencias combinadas asociadas con síndromes (n = 5). Además, se identificó un paciente con falla medular, otro con desregulación inmunitaria y dos con inmunodeficiencia no grave que afectaba la inmunidad celular y humoral. De los 17 pacientes analizados, a 13 se les practicó estudio genético y 12 resultaron con alteraciones relacionadas con su fenotipo. Trece pacientes recibieron tratamiento con inmunoglobulina humana inespecífica intravenosa y tres fallecieron (cuadro 1).

En los 17 pacientes estudiados, el lóbulo inferior fue el segmento más frecuentemente afectado por bronquiectasias (n = 12), seguido del lóbulo superior y la língula en (n = 2). Respecto a la distribución de las bronquiectasias, se encontró que fue unilateral en 11 pacientes y bilateral en 5 (cuadro 3).

**Cuadro 3:** Características de las bronquiectasias (N = 17)

**Bronquiectasias**		n
	Bilaterales	5
	Unilaterales	11
	Sin dato	1
Ubicación		
	Língula	2
	Lóbulo inferior	12
	Lóbulo inferior y língula	1
	Lóbulo superior	2

## Discusión

Las bronquiectasias pueden ser una manifestación importante de los errores innatos de la inmunidad y actualmente se consideran un signo de alarma. La prevalencia estimada en este estudio fue del 4,6 % (17 pacientes), mucho más baja que la encontrada en el 2016 en Bogotá ^16^. La aparición temprana de bronquiectasias puede orientar hacia una sospecha diagnóstica, dado que los errores innatos de la inmunidad asociados con deficiencias de anticuerpos pueden manifestarse en las etapas tempranas de la vida ^1^^,^^8^. En el presente estudio, la edad media de los pacientes fue de nueve años, con un RIC de 5 a 12 años, lo que corrobora la afirmación previa y subraya la importancia de una evaluación temprana del sistema inmunológico en estos pacientes.

Los errores innatos de la inmunidad más frecuentemente identificados en la cohorte de pacientes estudiada fueron la inmunodeficiencia con predominio de defectos de los anticuerpos, seguida de las inmunodeficiencias combinadas asociadas con síndromes. Estos hallazgos son congruentes con lo descrito en la literatura ^2^^,^^8^. Además, en el estudio de Zea-Vera *et al*. -que incluyó pacientes adultos de Colombia con bronquiectasias no relacionadas con fibrosis quística o con neumonía recurrente-, también se encontró que las deficiencias predominantemente de anticuerpos fueron el error innato de la inmunidad más frecuente ^18^.

Las inmunodeficiencias combinadas asociadas con características sindrómicas fueron representativas, pues se encontraron en cinco pacientes. Esto reafirma que algunos síndromes genéticos complejos tienen un papel importante en la patogénesis de los errores innatos de la inmunidad y las bronquiectasias ^8^. En algunos estudios, se ha reportado el síndrome de Down y la ataxia telangiectasia como preponderantes ^19^. En la población de este estudio, dos pacientes tenían síndrome de Down. Actualmente, se ha documentado que el síndrome de Down es la alteración genética más común relacionada con los defectos inmunológicos, ya que cursa con desregulación inmunológica y alteraciones en la inmunidad innata y adaptativa, lo que puede impactar la función y la cantidad de los anticuerpos producidos. Los factores mencionados también se han implicado en el desarrollo de bronquiectasias ^20^.

Los casos de falla medular y de inmunodeficiencia, que afectan la inmunidad celular y humoral, fueron menos frecuentes, pero indican que puede haber diversos mecanismos inmunológicos implicados. En el metaanálisis de Brower *et al*. ^21^, en el que se evaluaron 12 estudios con un total de 989 niños con bronquiectasias no relacionadas con fibrosis quística, se encontró que en los 160 pacientes con bronquiectasias y errores innatos de la inmunidad, los trastornos de las células B estuvieron presentes en 117 pacientes. Las deficiencias de IgG y sus subclases fueron las más comunes (n = 106), y la deficiencia de IgA correspondió a 10 pacientes. Se encontró un grupo heterogéneo de trastornos de inmunodeficiencia combinada en 16 pacientes, mientras que en 12 pacientes se debió a trastornos de las células T. Estos resultados son similares a los encontrados en el presente estudio.

Por el contrario, en la investigación de Bekir *et al*., en la que evaluaron 74 pacientes de Turquía con bronquiectasias no relacionadas con fibrosis quística, se encontró que solo 23 (37 %) pacientes presentaron alguna alteración en la inmunidad humoral ^22^. Sin embargo, estos resultados podrían deberse a diferencias poblacionales y metodológicas, por lo que es difícil compararlos con los hallazgos del presente estudio. En este grupo se encontró que 56 pacientes presentaron alteraciones en la inmunidad humoral y 26 las tenía en las células T. Las causas de los errores innatos de la inmunidad encontradas en este grupo de pacientes, sugieren que la evaluación inicial mediante estudios, como hemograma completo (enfocado en la búsqueda de linfopenias u otras alteraciones celulares), inmunoglobulinas séricas, subclases de IgG y reacciones a antígenos vacunales, son una estrategia útil para el estudio de pacientes pediátricos con bronquiectasias sin causa evidente ^23^.

El estudio genético realizado en 13 de nuestros pacientes reveló que 12 tenían alteraciones genéticas relacionadas con su fenotipo clínico, lo cual es coherente con la creciente evidencia de que las pruebas genéticas pueden identificar mutaciones relacionadas con errores innatos de la inmunidad, infecciones recurrentes y desarrollo de bronquiectasias con pérdida de parénquima pulmonar ^1^^,^^2^^,^^8^^,^^19^.

En nuestro grupo de pacientes, la afectación unilateral fue más común (n = 11) en comparación con la multilobar (n = 5). Este hallazgo contrasta con lo reportado en la literatura, es decir, que las bronquiectasias multilobares son más frecuentes en los pacientes con errores innatos de la inmunidad ^14^. Esto podría reflejar variaciones particulares en la presentación clínica, edad temprana de diagnóstico y progresión de la enfermedad. También, se observó que las bronquiectasias afectaron el lóbulo inferior en 12 pacientes y, al lóbulo superior y a la língula, en 2 pacientes cada uno. Estos hallazgos coinciden con otros estudios en los cuales es más frecuente el compromiso del lóbulo medio o el inferior ^1^^,^^2^^,^^12^^,^^15^.

Se administró inmunoglobulina humana inespecífica intravenosa a 13 pacientes, lo que concuerda con lo establecido en la literatura científica como tratamiento para estos tipos de defectos inmunológicos ^24^^,^^25^. Este suplemento se indicó en algunas inmunodeficiencias humorales y otras combinadas, ya que ha demostrado reducir la frecuencia de infecciones respiratorias y disminuir la desregulación inmunológica, lo que permite estabilizar la función pulmonar ^24^^,^^25^. Además, 3 pacientes fallecieron, lo que resalta la necesidad de una intervención individualizada temprana.

La inmunodeficiencia con predominio de defectos de los anticuerpos, seguida de las inmunodeficiencias combinadas asociadas con características sindrómicas, fueron los errores innatos de la inmunidad que presentaron con mayor frecuencia bronquiectasias no relacionadas con fibrosis quística. La mortalidad observada y la variabilidad de los perfiles inmunológicos resaltan la necesidad de un diagnóstico oportuno y de intervenciones adecuadas que mejoren el pronóstico y la supervivencia de estos pacientes. Es fundamental un enfoque diagnóstico y terapéutico integral que incluya estudios genéticos y tratamiento con inmunoglobulina humana inespecífica intravenosa cuando esté indicada, para optimizar el manejo y el pronóstico de estos pacientes.
